# Hexaaqua­dibromidoeuropium(III) bromide, [EuBr_2_(H_2_O)_6_]Br

**DOI:** 10.1107/S1600536808014359

**Published:** 2008-05-17

**Authors:** Constantin Hoch, Arndt Simon

**Affiliations:** a Max-Planck-Institut für Festkörperforschung, Heisenbergstrasse 1, D-70569 Stuttgart, Germany

## Abstract

The title compound crystallizes with the GdCl_3_·6H_2_O structure type, exhibiting discrete [EuBr_2_(H_2_O)_6_]^+^ cations as the main building blocks, linked with isolated bromide anions *via* H⋯Br hydrogen bonds to form a complex framework. The Eu atom and one Br atom each lie on a twofold rotation axis.

## Related literature

For related literature, see: Bärnighausen *et al.* (1965 ▶); Bell & Smith (1990 ▶); Burns & Peterson (1971 ▶); Demyanets *et al.* (1974 ▶); Duhlev *et al.* (1988 ▶); Graeber *et al.* (1966 ▶); Habenschuss & Spedding (1980 ▶); Junk *et al.* (1999 ▶); Kolitsch (2006 ▶); Marezio *et al.* (1961 ▶); Reuter *et al.* (1994 ▶); Tegenfeldt *et al.* (1979 ▶); Wickleder & Meyer (1995 ▶).

## Experimental

### 

#### Crystal data

[EuBr_2_(H_2_O)_6_]Br
*M* *_r_* = 499.79
Monoclinic, 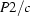

*a* = 8.1672 (7) Å
*b* = 6.7538 (4) Å
*c* = 12.5451 (10) Å
β = 127.077 (5)°
*V* = 552.08 (8) Å^3^
*Z* = 2
Mo *K*α radiation
μ = 16.52 mm^−1^
*T* = 293 (2) K
0.25 × 0.24 × 0.18 mm

#### Data collection

Stoe IPDSII diffractometer
Absorption correction: numerical [*X-RED* (Stoe & Cie, 2001 ▶) and *X-SHAPE* (Stoe & Cie, 1999 ▶)] *T*_min_ = 0.065, *T*_max_ = 0.155
10921 measured reflections
1613 independent reflections
1397 reflections with *I* > 2s(*I*)
*R*_int_ = 0.067

#### Refinement

*R*[*F* ^2^ > 2σ(*F* ^2^)] = 0.028
*wR*(*F* ^2^) = 0.049
*S* = 1.11
1613 reflections
72 parameters
All H-atom parameters refined
Δρ_max_ = 1.14 e Å^−3^
Δρ_min_ = −1.10 e Å^−3^

### 

Data collection: *X-AREA* (Stoe & Cie, 2006 ▶); cell refinement: *X-AREA*; data reduction: *X-AREA*; program(s) used to solve structure: *SHELXS97* (Sheldrick, 2008 ▶); program(s) used to refine structure: *SHELXL97* (Sheldrick, 2008 ▶); molecular graphics: *DRAWXTL* (Finger *et al.*, 2007 ▶); software used to prepare material for publication: *PLATON* (Spek, 2003 ▶).

## Supplementary Material

Crystal structure: contains datablocks global, I. DOI: 10.1107/S1600536808014359/mg2051sup1.cif

Structure factors: contains datablocks I. DOI: 10.1107/S1600536808014359/mg2051Isup2.hkl

Additional supplementary materials:  crystallographic information; 3D view; checkCIF report

## Figures and Tables

**Table:** 

Eu1—Br1	2.9449 (5)
Eu1—O1	2.424 (3)
Eu1—O2	2.422 (3)
Eu1—O3	2.388 (3)

**Table:** 

Br1—Eu1—O1	146.89 (8)
Br1—Eu1—O1^i^	76.21 (9)
Br1—Eu1—O2	77.33 (8)
Br1—Eu1—O2^i^	78.22 (8)
Br1—Eu1—O3	107.21 (9)
Br1—Eu1—O3^i^	143.18 (8)
Br1—Eu1—Br1^i^	84.41 (2)
O1—Eu1—O1^i^	132.3 (2)
O2—Eu1—O2^i^	146.8 (2)
O3—Eu1—O3^i^	84.5 (2)
O1—Eu1—O2	72.6 (1)
O1—Eu1—O2^i^	122.0 (1)
O1—Eu1—O3	75.8 (1)
O1—Eu1—O3^i^	69.3 (1)
O2—Eu1—O3	70.9 (1)
O2—Eu1—O3^i^	138.6 (1)

Symmetry code: (i) 
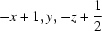
.

**Table 2:** Hydrogen-bond geometry (Å, °)

*D*—H⋯*A*	*D*—H	H⋯*A*	*D*⋯*A*	*D*—H⋯*A*
O1—H11⋯Br2^ii^	0.83 (2)	2.53 (8)	3.343 (4)	168 (6)
O1—H12⋯Br1^iii^	0.83 (2)	2.52 (13)	3.333 (4)	165 (6)
O2—H21⋯Br1^iv^	0.82 (2)	2.49 (10)	3.307 (4)	172 (6)
O2—H22⋯Br2^v^	0.83 (2)	2.63 (11)	3.417 (4)	161 (6)
O3—H31⋯Br1^vi^	0.83 (2)	2.46 (8)	3.288 (4)	173 (6)
O3—H32⋯Br2	0.83 (2)	2.52 (11)	3.328 (5)	163 (6)

Symmetry codes: (ii) 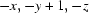; (iii) 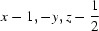; (iv) 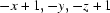; (v) 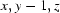; (vi) 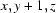.

**Fig. 1.**
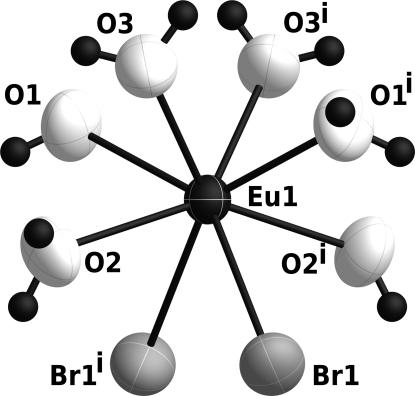
View of the cationic [Eu(H2O)6Br2]+ unit in [Eu(H2O)6Br2]Br, with displacement ellipsoids drawn at the 90% propability level. H atoms are shown as black spheres of arbitrary radii. [Symmetry code: (i) -x, y, 1/2 - z.]

**Fig. 2.**
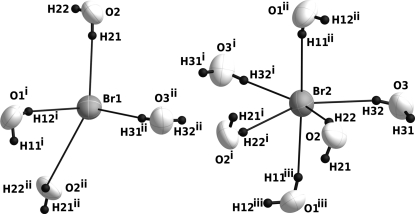
View of the H—Br contacts in [Eu(H2O)6Br(2)2]Br(1), left: four hydrogen bonds link Br1 to water molecules, right: six hydrogen bonds link Br2 to water molecules. All displacement ellipsoids are drawn at the 90% propability level. [Symmetry codes: (i) -x, y, 1/2 - z; (ii) -x, -y, -z; (iii) x, -y, 1/2 + z.]

**Fig. 3.**
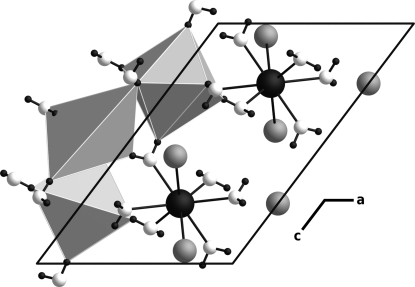
View along (010) on the crystal structure of [Eu(H2O)6Br2]Br. Small black spheres represent H atoms, large black spheres represent Eu atoms, grey spheres represent Br atoms, light grey spheres represent O atoms. Grey polyhedra represent the coordination of H atoms around Br atoms.

## supplementary crystallographic information

### Comment

[EuBr_2_(H_2_O)_6_]Br crystallizes in the monoclinic space group P2/c (No. 13) and is isotypic with the GdCl_3_^.^6H_2_O structure type (Marezio *et al.*, 1961), like many chloride hexahydrates MCl_3_^.^6H_2_O with *M* = Y (Bell & Smith, 1990), Ce (Reuter *et al.*, 1994), Nd (Habenschuss & Spedding, 1980), Sm - Tm (Graeber *et al.*, 1966), Am, Bk (Burns & Peterson, 1971), and two bromide hexahydrates MBr_3_^.^6H_2_O (*M* = Pr, Dy, Junk *et al.*, 1999).

The Eu atoms in [EuBr_2_(H_2_O)_6_]Br are coordinated by six water molecules and two bromine atoms forming a distorted square antiprism (Fig. 1). Hydrogen bonds H—Br connect the [EuBr_2_(H_2_O)_6_]^+^ cations with the Br^-^ counter-anions to a network. The bromine atom Br1 belonging to the cationic complex is surrounded by four, the isolated bromine atom Br2 by six hydrogen bonds (Fig. 2). A view of the unit cell of [EuBr_2_(H_2_O)_6_]Br is given in Fig. 3.

The H—Br distances (2.46–2.63 Å) are in good agreement with those in other bromide hydrates (e.g. 2.38–2.54 Å in [Sc(H_2_O)_5_(OH)]Br_2_, Kolitsch, 2006; 2.32–2.80 Å in [Ca(H_2_O)_6_]_2_[CdBr_6_], Duhlev *et al.*, 1988; 2.40–2.83 A in NaBr^.^2H_2_O, Tegenfeldt *et al.*, 1979). The Eu^III^—O distances in [EuBr_2_(H_2_O)_6_]Br range from 2.39 to 2.42 Å and thus are very similar to those in EuCl_3_^.^3H_2_O (2.39–2.40 Å, Reuter *et al.*, 1994), EuCl_3_^.^6H_2_O (2.39–2.43 Å, Graeber *et al.*, 1966), or EuCl(OH)_2_ (2.35–2.44 Å, Demyanets *et al.*, 1974). The same holds for the Eu^III^—Br distances in [EuBr_2_(H_2_O)_6_]Br (2.94 Å) which lie between those in Na_3_EuBr_6_ (2.83 Å, Wickleder & Meyer, 1995) and those in EuOBr (3.19 Å, Bärnighausen *et al.*, 1965).

### Experimental

Colourless single crystals of [EuBr_2_(H_2_O)_6_]Br were obtained by recrystallizing the commercially available product ("EuBr_3_^.^*X* H_2_O", Alfa Aesar, 99.99%) under argon from degassed aqueous HBr solution by slow cooling of a solution saturated at *ca* 60 °C to room temperature.

### Refinement

The positions of all hydrogen atoms were identified from the difference Fourier map, close to their ideal positions. Their refinement was performed applying a *DFIX* command (Sheldrick, 2008), restricting the O—H bond lengths to 0.82 ± 0.02 Å.

### Crystal data

**Table:**

[EuBr_2_(H_2_O)_6_]Br	*F*_000_ = 456
*M**_r_* = 499.79	*D*_x_ = 3.006 Mg m^−^^3^
Monoclinic, *P*2/*c*	Mo *K*α radiation λ = 0.71073 Å
Hall symbol: -P 2yc	Cell parameters from 10367 reflections
*a* = 8.1672 (7) Å	θ = 3.0–32.1º
*b* = 6.7538 (4) Å	µ = 16.52 mm^−^^1^
*c* = 12.5451 (10) Å	*T* = 293 (2) K
β = 127.077 (5)º	Irregular polyhedron, clear colourless
*V* = 552.08 (8) Å^3^	0.25 × 0.24 × 0.18 mm
*Z* = 2	

### Data collection

**Table:**

Stoe IPDSII diffractometer	1613 independent reflections
Radiation source: fine-focus sealed tube	1397 reflections with *I* > 2s(*I*)
Monochromator: graphite	*R*_int_ = 0.067
*T* = 293(2) K	θ_max_ = 30.0º
ω scans (in two runs with φ_1_ = 0° and φ_2_ = 90°)	θ_min_ = 3.0º
Absorption correction: numerical[X-RED (Stoe & Cie, 2001) and X-SHAPE (Stoe & Cie, 1999)]	*h* = −11→11
*T*_min_ = 0.065, *T*_max_ = 0.155	*k* = −9→9
10921 measured reflections	*l* = −17→17

### Refinement

**Table:**

Refinement on *F*^2^	Hydrogen site location: difference Fourier map
Least-squares matrix: full	All H-atom parameters refined
*R*[*F*^2^ > 2σ(*F*^2^)] = 0.028	*w* = 1/[σ^2^(*F*_o_^2^) + (0.0169*P*)^2^] where *P* = (*F*_o_^2^ + 2*F*_c_^2^)/3
*wR*(*F*^2^) = 0.049	(Δ/σ)_max_ < 0.001
*S* = 1.11	Δρ_max_ = 1.14 e Å^−^^3^
1613 reflections	Δρ_min_ = −1.10 e Å^−^^3^
72 parameters	Extinction correction: SHELXL97 (Sheldrick, 2008), Fc^*^=kFc[1+0.001xFc^2^λ^3^/sin(2θ)]^-1/4^
Primary atom site location: structure-invariant direct methods	Extinction coefficient: 0.0409 (10)
Secondary atom site location: difference Fourier map	

### Special details

**Table:**

Experimental. The title compoud is a commercially available chemical (Alfa Aesar) and was recrystallized under argon from degassed aqueous HBr solution. A suitable single-crystal was sealed with mother liquor in a thin-walled glass capillary.
Geometry. All e.s.d.'s (except the e.s.d. in the dihedral angle between two l.s. planes) are estimated using the full covariance matrix. The cell e.s.d.'s are taken into account individually in the estimation of e.s.d.'s in distances, angles and torsion angles; correlations between e.s.d.'s in cell parameters are only used when they are defined by crystal symmetry. An approximate (isotropic) treatment of cell e.s.d.'s is used for estimating e.s.d.'s involving l.s. planes.
Refinement. Refinement of *F*^2^ against ALL reflections. The weighted *R*-factor *wR* and goodness of fit *S* are based on *F*^2^, conventional *R*-factors *R* are based on *F*, with *F* set to zero for negative *F*^2^. The threshold expression of *F*^2^ > σ(*F*^2^) is used only for calculating *R*-factors(gt) *etc*. and is not relevant to the choice of reflections for refinement. *R*-factors based on *F*^2^ are statistically about twice as large as those based on *F*, and *R*- factors based on ALL data will be even larger.

### Fractional atomic coordinates and isotropic or equivalent isotropic displacement parameters (Å^2^)

**Table:**

	*x*	*y*	*z*	*U*_iso_*/*U*_eq_	
Eu1	0.50000	0.16454 (4)	0.25000	0.0170 (1)	
Br1	0.70613 (6)	−0.15845 (7)	0.44669 (4)	0.0295 (1)	
Br2	0.00000	0.63151 (9)	0.25000	0.0318 (2)	
O1	0.1772 (5)	0.3097 (5)	0.0676 (3)	0.0320 (7)	
O2	0.2413 (5)	0.0620 (5)	0.2757 (4)	0.0313 (7)	
O3	0.4434 (5)	0.4262 (5)	0.3524 (4)	0.0335 (7)	
H11	0.148 (11)	0.336 (10)	−0.006 (4)	0.06 (2)*	
H12	0.072 (9)	0.258 (14)	0.050 (10)	0.10 (3)*	
H21	0.253 (12)	0.098 (11)	0.342 (5)	0.07 (2)*	
H22	0.183 (11)	−0.046 (6)	0.253 (9)	0.09 (3)*	
H31	0.518 (10)	0.526 (7)	0.376 (8)	0.08 (2)*	
H32	0.321 (5)	0.456 (12)	0.315 (8)	0.09 (3)*	

### Atomic displacement parameters (Å^2^)

**Table:**

	*U*^11^	*U*^22^	*U*^33^	*U*^12^	*U*^13^	*U*^23^
Eu1	0.0168 (1)	0.0180 (1)	0.0172 (1)	0.000	0.0109 (1)	0.000
Br1	0.0295 (2)	0.0293 (2)	0.0266 (3)	0.0027 (2)	0.0152 (2)	0.0066 (2)
Br2	0.0294 (3)	0.0360 (3)	0.0327 (4)	0.000	0.0202 (3)	0.000
O1	0.0233 (13)	0.0385 (17)	0.0256 (18)	0.0036 (12)	0.0102 (13)	0.0084 (14)
O2	0.0316 (15)	0.0367 (17)	0.0355 (19)	−0.0076 (13)	0.0254 (15)	−0.0055 (14)
O3	0.0308 (15)	0.0302 (15)	0.042 (2)	−0.0035 (13)	0.0229 (16)	−0.0106 (14)

### Geometric parameters (Å, °)

**Table:**

Eu1—Br1	2.9449 (5)	Eu1—O3^i^	2.388 (3)
Eu1—Br1^i^	2.9449 (5)	O1—H11	0.82 (2)
Eu1—O1	2.424 (3)	O1—H12	0.83 (2)
Eu1—O1^i^	2.424 (3)	O2—H21	0.82 (2)
Eu1—O2	2.422 (3)	O2—H22	0.82 (2)
Eu1—O2^i^	2.422 (3)	O3—H31	0.83 (2)
Eu1—O3	2.388 (3)	O3—H32	0.83 (2)
			
Br1—Eu1—O1	146.89 (8)	O1—Eu1—O2	72.6 (1)
Br1^i^—Eu1—O1^i^	146.89 (8)	O1^i^—Eu1—O2^i^	72.6 (1)
Br1—Eu1—O1^i^	76.21 (9)	O1—Eu1—O2^i^	122.0 (1)
Br1^i^—Eu1—O1	76.21 (9)	O1^i^—Eu1—O2	122.0 (1)
Br1—Eu1—O2	77.33 (8)	O1—Eu1—O3	75.8 (1)
Br1^i^—Eu1—O2^i^	77.33 (8)	O1^i^—Eu1—O3^i^	75.8 (1)
Br1—Eu1—O2^i^	78.22 (8)	O1—Eu1—O3^i^	69.3 (1)
Br1^i^—Eu1—O2	78.22 (8)	O1^i^—Eu1—O3	69.3 (1)
Br1—Eu1—O3	107.21 (9)	O2—Eu1—O3	70.9 (1)
Br1^i^—Eu1—O3^i^	107.21 (9)	O2^i^—Eu1—O3^i^	70.9 (1)
Br1—Eu1—O3^i^	143.18 (8)	O2—Eu1—O3^i^	138.6 (1)
Br1^i^—Eu1—O3	143.18 (8)	O2^i^—Eu1—O3	138.6 (1)
Br1—Eu1—Br1^i^	84.41 (2)	H11—O1—H12	104 (8)
O1—Eu1—O1^i^	132.3 (2)	H21—O2—H22	107 (8)
O2—Eu1—O2^i^	146.8 (2)	H31—O3—H32	112 (8)
O3—Eu1—O3^i^	84.5 (2)		

Symmetry codes: (i) −*x*+1, *y*, −*z*+1/2.

### Hydrogen-bond geometry (Å, °)

**Table:**

*D*—H···*A*	*D*—H	H···*A*	*D*···*A*	*D*—H···*A*
O1—H11···Br2^ii^	0.83 (2)	2.53 (8)	3.343 (4)	168 (6)
O1—H12···Br1^iii^	0.83 (2)	2.52 (13)	3.333 (4)	165 (6)
O2—H21···Br1^iv^	0.82 (2)	2.49 (10)	3.307 (4)	172 (6)
O2—H22···Br2^v^	0.83 (2)	2.63 (11)	3.417 (4)	161 (6)
O3—H31···Br1^vi^	0.83 (2)	2.46 (8)	3.288 (4)	173 (6)
O3—H32···Br2	0.83 (2)	2.52 (11)	3.328 (5)	163 (6)

Symmetry codes: (ii) −*x*, −*y*+1, −*z*; (iii) *x*−1, −*y*, *z*−1/2; (iv) −*x*+1, −*y*, −*z*+1; (v) *x*, *y*−1, *z*; (vi) *x*, *y*+1, *z*.
